# Supplementary material for: Phonophoresis through Nonsteroidal Anti-Inflammatory Drugs for Knee Osteoarthritis Treatment: Systematic Review and Meta-Analysis
Source: Biomedicines. 2022 Dec 14;10(12):3254. doi: 10.3390/biomedicines10123254 (PMC9775989; doi:10.3390/biomedicines10123254)
Supplement: Supplementary file 1 [file biomedicines-10-03254-s001.zip › Supplementary Table S1.pdf]

Supplementary Table S1. Search strategy.

| WEB OF SCIENCE | Results |
|---|---|
| *Phonophoresis AND Drugs* | 123 |
| *Phonophoresis AND Ions* | 3 |
| *Phonophoresis AND Inflammation* | 22 |
| *Phonophoresis AND Pharmaceutical preparations* | 4 |
| TOTAL | 152 |

| SCOPUS | Results |
|---|---|
| *Phonophoresis AND Drugs* | 107 |
| *Phonophoresis AND Ions* | 1 |
| *Phonophoresis AND Inflammation* | 20 |
| *Phonophoresis AND Pharmaceutical preparations* | 3 |
| TOTAL | 131 |

| PUBMED | Results |
|---|---|
| *Phonophoresis AND Drugs* | 43 |
| *Phonophoresis AND Ions* | 3 |
| *Phonophoresis AND Inflammation* | 14 |
| *Phonophoresis AND Pharmaceutical preparations* | 30 |
| TOTAL | 90 |

| CINAHL Complete | Results |
|---|---|
| *Phonophoresis AND Drugs* | 26 |
| *Phonophoresis AND Ions* | 1 |
| *Phonophoresis AND Inflammation* | 5 |
| *Phonophoresis AND Pharmaceutical preparations* | 0 |
| TOTAL | 32 |

| SciELO | Results |
|---|---|
| *Phonophoresis AND Drugs* | 2 |
| *Phonophoresis AND Ions* | 0 |
| *Phonophoresis AND Inflammation* | 3 |
| *Phonophoresis AND Pharmaceutical preparations* | 0 |
| TOTAL | 5 |

| PEDro | Results |
|---|---|
| *Phonophoresis AND Drugs* | 0 |
| *Phonophoresis AND Ions* | 0 |
| *Phonophoresis AND Inflammation* | 1 |
| *Phonophoresis AND Pharmaceutical preparations* | 0 |
| TOTAL | 1 |
